# Psychosocial and Behavioral Factors Associated with Excessive Smartphone Use Among Korean Adolescents: A National Cross-Sectional Study

**DOI:** 10.3390/children13040472

**Published:** 2026-03-28

**Authors:** So Ra Kang

**Affiliations:** College of Nursing, Wonkwang University, 460 Iksandae-ro, Iksan, Jeonbuk-do 54538, Republic of Korea; srkang01@wku.ac.kr

**Keywords:** adolescents, behavioral health, digital health, excessive smartphone use, psychosocial factors

## Abstract

**Highlights:**

**What are the main findings?**

- In a nationally representative sample of Korean adolescents, excessive smartphone use was common and associated with developmental, socioeconomic, and psychological factors.
- Female sex, higher school grade, lower perceived socioeconomic status, anxiety symptoms, suicidal ideation, poorer sleep-related recovery, and vigorous physical activity were associated with higher odds of excessive smartphone use.

**What are the implications of the main findings?**

- Excessive smartphone use among adolescents appears to be embedded within broader developmental, social, and psychological contexts.
- These findings highlight the importance of considering developmental stage, social environment, and psychological factors when addressing adolescent digital health.

**Abstract:**

**Background/Objectives**: Excessive smartphone use has emerged as an important behavioral health concern during adolescence, a developmental period characterized by heightened psychosocial vulnerability. This study aimed to identify psychosocial and behavioral factors associated with excessive smartphone use among Korean adolescents using nationally representative data. **Methods**: Data were obtained from the 2024 Korean Youth Risk Behavior Web-based Survey (KYRBS), including 54,653 adolescents. Excessive smartphone use was operationally defined as average daily smartphone use of ≥300 min. Multivariable logistic regression was conducted to examine associated factors. An exploratory machine learning analysis using a Light Gradient Boosting Machine included 52,450 participants with complete predictor data. **Results**: Female sex, higher grade level, lower perceived socioeconomic status, higher perceived daily stress, higher anxiety symptoms, poorer sleep-related recovery, suicidal ideation, and more frequent vigorous physical activity were associated with higher odds of excessive smartphone use. The supplementary modeling approach showed patterns consistent with the regression findings, with grade level, socioeconomic status, and sex contributing prominently. Vigorous physical activity demonstrated a nonlinear association with predicted risk. **Conclusions**: Excessive smartphone use among adolescents appears to be shaped by developmental stage, socioeconomic context, and psychological vulnerability. These findings support prevention strategies that address emotional well-being and sleep health alongside broader structural and school-based approaches.

## 1. Introduction

Adolescence is a critical developmental period characterized by rapid biological, psychological, and social transitions [1,2]. During this stage, health-related behaviors are established and may shape long-term health trajectories across the life course [3]. In contemporary societies, smartphones have become embedded in adolescents’ daily environments, serving educational, communicative, and recreational functions [4,5]. Given the developmental sensitivity of this age group, patterns of digital media use may have implications for behavioral health and population well-being.

Excessive smartphone use generally refers to prolonged or high-duration engagement with smartphones, typically quantified by daily use time. While excessive use does not necessarily imply clinical dependence, excessive or problematic smartphone use has been described as involving impaired control and functional difficulties in daily life [6,7]. A growing body of research has linked problematic smartphone use to emotional symptoms, depressive features, and attentional difficulties [8,9,10], as well as sleep disturbances and related health concerns [11,12,13]. National survey–based studies have further identified sociodemographic and behavioral correlates, including sex differences, lifestyle factors, and physical activity patterns [14,15]. However, findings across studies have not always been consistent, partly due to differences in measurement approaches, population characteristics, and analytic strategies. While some researchers emphasize overall screen time exposure as the primary risk factor [11,12], others suggest that psychosocial vulnerability and contextual factors may play a more substantial role in adverse outcomes [16,17]. However, the extent to which duration alone reflects problematic use remains debated. Accordingly, examining excessive smartphone use as a time-based exposure at the population level may help clarify how developmental and contextual factors are associated with digital behavior patterns in adolescence.

Beyond overall screen exposure, recent research has increasingly emphasized that adolescent digital behavior is closely intertwined with emotional regulation processes and mental health outcomes [18,19]. Smartphones provide adolescents with continuous access to social interaction, entertainment, and information, which may influence how young people cope with daily stressors and regulate emotions [20]. For some adolescents, digital engagement may function as a strategy for mood management, distraction, or social reassurance in response to negative emotional states [21]. However, emerging evidence suggests that heavy or dysregulated digital media use may also reinforce maladaptive coping patterns and contribute to emotional vulnerability. Studies in adolescent populations have reported associations between intensive digital media use and depressive symptoms, anxiety, emotional dysregulation, and reduced well-being [22,23]. At the same time, researchers increasingly caution that digital behavior should be understood within a broader developmental and psychosocial context rather than interpreted solely as a harmful exposure [24,25,26,27]. Accordingly, examining excessive smartphone use alongside psychosocial and behavioral factors may provide a more comprehensive understanding of adolescent digital health.

From a population health perspective, these developmental and contextual factors may shape patterns of smartphone use across adolescent populations. Grade level may reflect variations in academic pressure, peer relationships, and autonomy, whereas perceived socioeconomic status may capture disparities in family resources and social context [1,2]. Socioeconomic gradients in adolescent health behaviors are well documented [3], and examining such gradients in relation to excessive smartphone use may help identify subpopulations at increased risk. The use of nationally representative data enables assessment of population-level patterns and strengthens the generalizability of findings.

In South Korea, smartphone penetration among adolescents is highly prevalent, consistent with broader global trends in digital engagement, and nationally representative surveillance systems provide comprehensive population-based data on adolescent health behaviors [4,5,14]. Despite prior evidence linking smartphone use to mental health indicators [8,9,10,11,12,13], comparatively less attention has been given to examining excessive smartphone use as a time-based exposure in relation to developmental, socioeconomic, and behavioral factors within nationally representative adolescent populations.

Therefore, this study aimed to identify psychosocial and behavioral factors associated with excessive smartphone use among Korean adolescents using national survey data. Multivariable logistic regression and a complementary machine learning approach were applied to examine associations and potential nonlinear patterns. This investigation provides population-level insight into developmental and contextual correlates of adolescent smartphone use.

## 2. Materials and Methods

### 2.1. Study Design and Data Source

This cross-sectional study used data from the 2024 Korea Youth Risk Behavior Web-Based Survey (KYRBS), an annual nationally representative survey conducted by the Korea Disease Control and Prevention Agency (KDCA). The KYRBS employs a stratified, multistage cluster sampling design to obtain representative data on health behaviors, mental health, and sociodemographic characteristics among Korean middle and high school students. Schools are selected as primary sampling units, and classes are randomly sampled within schools. Sampling weights are provided to allow population-level inference.

The survey is anonymous and self-administered during school hours under standardized procedures. The KYRBS was approved by the Institutional Review Board of the KDCA, and informed consent was obtained from participants in accordance with national ethical guidelines. This study analyzed publicly available, de-identified secondary data and complied with relevant ethical standards.

### 2.2. Participants

Adolescents who participated in the 2024 KYRBS and had available information on smartphone use time and study variables were included in the analysis. Participants with missing or implausible values for smartphone use time were excluded. After applying complete-case criteria, 54,653 adolescents were included in the multivariable logistic regression analysis. For exploratory modeling requiring complete data across all predictors, listwise deletion resulted in a final analytic sample of 52,450 adolescents.

### 2.3. Measures

#### 2.3.1. Excessive Smartphone Use

In this study, excessive smartphone use was operationally defined as average daily smartphone use of ≥300 min (5 h). Previous large-scale studies have reported that mental health indicators, including depressive symptoms, anxiety, and suicidal ideation, tend to worsen beyond approximately five hours of daily screen exposure, suggesting a potential threshold effect [28,29]. In addition, considering the structured daily schedule of Korean adolescents, prolonged smartphone use beyond this duration may displace essential developmental activities such as sleep and physical activity [30]. Therefore, this threshold was used as a pragmatic population-level indicator of higher-duration exposure rather than a clinical cutoff for behavioral addiction or functional impairment. Importantly, this operational definition does not distinguish between high engagement and problematic or maladaptive smartphone use, and should be interpreted as reflecting higher-duration exposure rather than pathological behavior. Those reporting <300 min per day were classified as the comparison group. Sensitivity analyses were additionally conducted using alternative thresholds of 240 and 360 min per day to assess robustness.

#### 2.3.2. Sociodemographic Characteristics

Sociodemographic variables included sex (male/female), grade (grades 7–12, corresponding to middle and high school levels), and perceived socioeconomic status (SES). Perceived SES was assessed on a five-point scale (1 = high to 5 = low), with higher scores indicating lower socioeconomic position.

#### 2.3.3. Mental Health Variables

Perceived stress was assessed using a five-point Likert scale (1 = very much to 5 = not at all). Anxiety symptoms were measured using the Generalized Anxiety Disorder-7 (GAD-7) scale. Total scores were calculated by summing the seven items, with higher scores indicating greater anxiety severity. Suicidal ideation during the past 12 months was assessed as a dichotomous variable (yes/no).

#### 2.3.4. Health Behaviors

Perceived recovery from sleep was measured on a five-point scale (1 = very sufficient to 5 = not sufficient at all), with higher scores indicating poorer sleep-related recovery.

Vigorous physical activity was assessed as the number of days (0–7) during the past week in which participants engaged in high-intensity physical activity.

### 2.4. Statistical Analysis

Descriptive statistics were calculated to summarize participant characteristics. Categorical variables are presented as frequencies and percentages, and continuous variables as means and standard deviations. Group differences between adolescents with and without excessive smartphone use were examined using chi-square tests for categorical variables and independent *t*-tests for continuous variables. Multivariable logistic regression analysis was conducted to identify factors independently associated with excessive smartphone use. All covariates were entered simultaneously to control for potential confounding. Adjusted odds ratios (aORs) and 95% confidence intervals (CIs) were estimated. Model fit was evaluated using the Hosmer–Lemeshow goodness-of-fit test and Nagelkerke’s R^2^. All analyses were performed using SPSS (version 29.0; IBM Corp., Armonk, NY, USA). Statistical significance was defined as a two-sided *p*-value < 0.05. Sensitivity analyses were conducted using alternative smartphone use thresholds.

### 2.5. Exploratory Modeling Approach

In addition to regression analysis, an exploratory machine learning approach was applied to examine potential nonlinear relationships between predictors and excessive smartphone use. The binary outcome variable was defined using the operational threshold described above (≥300 min per day). Smartphone use time variables were used only to define the outcome and were excluded from the set of predictors. The dataset was randomly divided into training (70%) and test (30%) sets using stratified sampling. A Light Gradient Boosting Machine model was trained on the training dataset. Model performance was evaluated using the area under the receiver operating characteristic curve (AUC). To improve interpretability, SHapley Additive exPlanations (SHAP) were used to explore the relative contribution and direction of predictors. This analysis was conducted as a complementary exploratory approach to support and extend the findings from the regression analysis, rather than to serve as a primary predictive model. Accordingly, it was intended to provide additional insights into variable importance and potential non-linear relationships, and does not replace regression-based inference.

## 3. Results

### 3.1. Participant Characteristics

The final analytic sample consisted of 54,653 Korean adolescents drawn from a nationally representative survey. Of these, 51.4% were male and 48.6% were female. Participants were relatively evenly distributed across grade levels from middle school year 1 to high school year 3 (grade 7–12). With respect to socioeconomic status, 46.5% reported middle perceived SES, followed by upper-middle (30.8%) and high (11.6%), whereas 11.1% reported lower-middle or low SES. Regarding perceived stress, 11.2% of participants reported very high stress and 30.9% high stress, whereas 15.8% reported low or very low stress. The mean GAD-7 score was 11.5 ± 4.7. Suicidal ideation during the past 12 months was reported by 12.7% of participants. Nearly half (47.1%) reported insufficient or very insufficient recovery from sleep. Adolescents engaged in vigorous physical activity on 3.3 ± 2.2 days per week. The mean daily smartphone use time was 304.2 ± 172.6 min (Table 1). Based on the predefined threshold of ≥300 min per day, the prevalence of excessive smartphone use was 42.8%.

### 3.2. Factors Associated with Excessive Smartphone Use

Multivariable logistic regression analysis was performed to examine factors independently associated with excessive smartphone use (Table 2).

Female adolescents had higher odds of excessive smartphone use compared with males (aOR = 1.44, 95% CI: 1.39–1.50, *p* < 0.001). Grade level showed a graded association. Compared with grade 7, the adjusted odds ratios were 1.45 (95% CI: 1.37–1.55) for grade 8, 1.84 (95% CI: 1.74–1.96) for grade 9, 1.60 (95% CI: 1.50–1.71) for grade 10, 2.00 (95% CI: 1.88–2.13) for grade 11, and 1.94 (95% CI: 1.82–2.07) for grade 12 (all *p* < 0.001), indicating increasing odds across school levels. Perceived SES demonstrated a socioeconomic gradient. Compared with high SES, adolescents reporting upper-middle (aOR = 1.11, 95% CI: 1.04–1.18, *p* = 0.002), middle (aOR = 1.52, 95% CI: 1.43–1.62, *p* < 0.001), lower-middle (aOR = 2.06, 95% CI: 1.90–2.23, *p* < 0.001), and low SES (aOR = 2.91, 95% CI: 2.52–3.36, *p* < 0.001) had progressively higher odds of excessive smartphone use.

Psychological factors were also associated with excessive smartphone use. Perceived daily stress score showed an inverse association with excessive smartphone use (aOR = 0.95, 95% CI: 0.93–0.97, *p* < 0.001). Because higher values represent lower perceived stress, this finding indicates that adolescents experiencing greater stress had higher odds of excessive smartphone use. GAD-7 score was incrementally associated with excessive smartphone use (aOR = 1.01 per one-point increase, 95% CI: 1.01–1.02, *p* < 0.001). Adolescents reporting suicidal ideation had higher odds compared with those without suicidal ideation (aOR = 1.20, 95% CI: 1.13–1.27, *p* < 0.001).

Sleep-related recovery was positively associated with excessive smartphone use (aOR = 1.04 per level increase, 95% CI: 1.02–1.06, *p* < 0.001), where higher scores indicated poorer recovery. Vigorous physical activity was modestly associated with higher odds (aOR = 1.02 per additional day, 95% CI: 1.01–1.03, *p* < 0.001).

The overall model was statistically significant (χ^2^ = 2478.95, df = 15, *p* < 0.001). The explained variance was modest (Cox & Snell R^2^ = 0.046; Nagelkerke R^2^ = 0.062), which is consistent with the multifactorial nature of adolescent behavioral outcomes.

### 3.3. Exploratory Modeling Analysis

An exploratory modeling analysis was conducted as a supplementary approach to examine predictive patterns. The model demonstrated modest discriminative performance (AUC ≈ 0.63). Consistent with the regression findings, grade level, perceived socioeconomic status, and sex were identified as influential variables in the modeling analysis (see Supplementary Figure S1). In addition, vigorous physical activity exhibited a nonlinear association with predicted probability of excessive smartphone use, indicating that higher levels of activity were not uniformly associated with lower predicted risk (see Supplementary Figure S2).

As this analysis was exploratory in nature, the primary interpretation of associations is based on the multivariable logistic regression results.

## 4. Discussion

This study examined sociodemographic and psychological factors associated with excessive smartphone use among Korean adolescents using nationally representative data. In this nationally representative sample of Korean adolescents, excessive smartphone use, defined using a time-based operational threshold, was commonly observed. In this study, the ≥300-min threshold was used as a pragmatic population-level indicator informed by prior evidence suggesting that adverse mental health outcomes may become more evident beyond approximately five hours of daily screen exposure [28,29], and is not intended to represent a clinical diagnostic cutoff for problematic smartphone use. It should be noted that the ≥300-min threshold represents a pragmatic, time-based indicator of exposure rather than a clinical or psychological diagnosis of problematic use.

Multivariable logistic regression analyses indicated that female sex, higher school grade, lower perceived socioeconomic status, elevated anxiety, poorer sleep-related recovery, and suicidal ideation were associated with higher odds of excessive smartphone use. These associations were observed across sociodemographic and psychological domains, suggesting that excessive smartphone use reflects multifactorial influences situated within developmental and social contexts. These findings can be interpreted within established theoretical frameworks, including the social determinants of health, coping behavior, and digital self-regulation perspectives. The exploratory modeling analysis yielded patterns consistent with the regression findings, supporting the stability of the observed associations. The machine learning analysis complemented the regression results by capturing potential non-linear relationships and providing insight into the relative importance of predictors. This complementary approach provides additional value by offering a data-driven assessment of variable importance. It also captures potential non-linear relationships that are not fully addressed in traditional regression models. In particular, the SHAP-based analysis enables an interpretable ranking of predictors, thereby enhancing the understanding of the relative contribution of key factors. Overall, these findings indicate that excessive smartphone use among adolescents is linked to interconnected developmental, social, and emotional contexts, and should be interpreted as reflecting patterns of exposure rather than indicating problematic or addictive use per se.

Female adolescents showed higher odds of excessive smartphone use compared with male adolescents. This finding is consistent with previous studies reporting greater engagement of girls in communication-oriented and social networking activities via smartphones [4,6,8]. Prior research has suggested that digital interaction may serve as an important avenue for peer connection and emotional exchange among adolescents [18,19,21]. Within this context, increased use may reflect gendered patterns of socialization and emotional coping in digital environments [19,21,24]. In addition, smartphone use among female adolescents may partly reflect emotionally oriented patterns of digital engagement, such as seeking reassurance, maintaining peer connectedness, or coping with interpersonal stress. Social expectations surrounding responsiveness, relational maintenance, and peer visibility may also contribute to more frequent or prolonged smartphone engagement in girls [18,19,21,24]. Taken together, these findings suggest that sex differences in smartphone use may be embedded within broader developmental and social dynamics.

Higher school grades were associated with greater odds of excessive smartphone use. Similar trends have been observed in previous epidemiological studies reporting increasing digital media engagement across adolescence [4,10]. From a developmental perspective, progression through middle and high school is accompanied by expanding social networks, increasing academic demands, and greater autonomy in daily decision-making [17]. As parental monitoring typically decreases and peer interaction becomes more central during later adolescence, smartphone use may assume a more prominent role in social communication and self-expression [1,3]. These patterns suggest that excessive smartphone use may reflect normative developmental transitions in autonomy and peer orientation.

Lower perceived socioeconomic status was associated with higher odds of excessive smartphone use. Previous research has identified socioeconomic gradients in digital behavior and adolescent health [3,5]. Disparities in parental monitoring, access to structured extracurricular activities, and availability of alternative leisure resources may influence usage patterns [5,27]. In addition, broader evidence indicates that media exposure and screen time are shaped by sociodemographic and environmental factors in children and adolescents [30,31], further supporting the role of contextual influences. In socially disadvantaged contexts, smartphones may represent a readily accessible form of entertainment or social participation [17,32]. Taken together, these findings suggest that smartphone use is situated within broader structural and socioeconomic conditions.

Higher levels of perceived daily stress were also associated with increased odds of excessive smartphone use. Adolescence is characterized by academic demands, peer-related pressures, and ongoing developmental transitions, all of which may contribute to heightened stress experiences [17]. Smartphones may function as an accessible means of distraction, social reassurance, or short-term stress relief in the context of everyday stressors. Previous research has noted that digital media use may intersect with adolescent well-being in complex and bidirectional ways [24], and associations between screen engagement and psychological distress have been reported in population-based studies [7,11,28]. Although the magnitude of the association observed in this study was modest, this finding suggests that routine stress experiences may contribute incrementally to digital engagement patterns. Given the cross-sectional design, the temporal direction of this relationship remains uncertain.

Elevated anxiety and the presence of suicidal ideation were associated with excessive smartphone use. Consistent with systematic reviews and national studies linking problematic smartphone use with depression and anxiety symptoms [6,7,8], adolescents experiencing emotional difficulties may engage in increased digital use as a means of distraction or mood regulation [19,21]. In addition, associations between screen time and depressive symptoms have been documented in adolescent populations [11]. However, the direction of this relationship remains uncertain given the cross-sectional design. These results highlight the interconnected nature of digital behavior and mental health during adolescence.

Poorer sleep-related recovery was also associated with excessive smartphone use. Previous research has documented associations between excessive smartphone use and sleep disturbance among adolescents [12,13]. Nighttime media engagement may interfere with sleep duration and quality, while insufficient sleep may in turn increase daytime digital engagement [19,21]. This reciprocal possibility underscores the relevance of digital hygiene and sleep health in adolescent populations.

More frequent vigorous physical activity was associated with higher odds of excessive smartphone use in the regression analysis. Although physical activity is generally associated with favorable health outcomes in adolescence, this finding suggests that smartphone use and physical activity should not necessarily be interpreted as oppositional behaviors. Instead, consistent with the concept of behavioral clustering, health-related behaviors in adolescence tend to co-occur within broader lifestyle patterns rather than operate independently [15,27].

From this perspective, physically active adolescents may also engage in high levels of smartphone use for social communication, coordination of activities, entertainment, or identity expression linked to peer groups and extracurricular participation [15]. These patterns may reflect increased social connectedness and activity density rather than sedentary or dysfunctional behavior alone. Furthermore, this finding can be understood within the framework of the social determinants of health, which emphasizes that adolescent behaviors are shaped by broader social, environmental, and contextual influences rather than isolated individual choices [3,27]. Access to peer networks, structured activities, and social environments may simultaneously promote both physical activity and digital engagement. Overall, these findings highlight the importance of interpreting smartphone use within the context of adolescents’ interconnected behavioral routines and social contexts, rather than as a single risk behavior.

Although the explanatory power of the model was modest (Nagelkerke R^2^ ≈ 0.06), relatively low R^2^ values are commonly observed in epidemiological studies, as examining complex adolescent health behaviors influenced by multiple social, psychological, and environmental factors [25,26]. Health-related behaviors during adolescence are shaped by multiple developmental, social, and environmental influences that cannot be fully captured by cross-sectional survey variables [27]. In addition, the association observed between poorer sleep-related recovery and excessive smartphone use may partially reflect interactions between sleep disruption and emotional regulation processes during adolescence [19,24]. Insufficient sleep and poor sleep quality have been associated with increased emotional reactivity and impaired emotional regulation [19,33], with similar patterns reported in clinical populations, which may contribute to greater engagement with digital media as a coping or distraction strategy.

Given the large sample size, many associations reached statistical significance; however, several effect sizes were modest in magnitude. This distinction is important when interpreting the findings, as statistical significance does not necessarily imply substantial real-world impact. For example, the associations observed for anxiety symptoms, sleep-related recovery, and vigorous physical activity were statistically significant but relatively small in magnitude, suggesting incremental rather than strong effects at the individual level. These findings should therefore be interpreted as reflecting population-level tendencies rather than strong predictive relationships for individual behavior. Although individual effect sizes were small, even modest associations may have meaningful implications at the population level given the high prevalence of smartphone use among adolescents. In contrast, variables such as school grade and perceived socioeconomic status demonstrated comparatively larger effect sizes, indicating more pronounced gradients across developmental and social contexts. Collectively, these results highlight the importance of considering both statistical significance and practical relevance when translating findings into policy or intervention strategies.

In addition to the regression analyses, the exploratory modeling approach identified similar sociodemographic and psychological characteristics—particularly school grade, sex, and perceived socioeconomic status—as prominent contributors. The overall pattern of associations was consistent with the regression findings. Notably, vigorous physical activity demonstrated a nonlinear association with predicted risk, suggesting that the relationship between activity levels and smartphone use may vary across different ranges of engagement [16]. In line with this finding, previous research suggests that health-related behaviors may cluster within broader lifestyle patterns, and physically active adolescents may also engage in higher levels of smartphone use [15,27]. As the modeling analysis was exploratory in nature and demonstrated modest discrimination, these results should be interpreted as supportive rather than confirmatory evidence.

The findings of this study indicate that excessive smartphone use is common among adolescents and is associated with developmental stage, socioeconomic context, and psychological characteristics. Rather than reflecting a single behavioral domain, excessive smartphone use appears to be situated within broader patterns of adolescent health and well-being.

The observed developmental and socioeconomic gradients suggest that prevention efforts may benefit from being tailored according to school grade and social context. Differences across grade levels highlight the importance of considering developmental transitions during adolescence, while socioeconomic variation underscores the role of structural and environmental conditions in shaping digital behavior. The associations with anxiety, suicidal ideation, and sleep-related recovery further suggest that smartphone use should be considered alongside mental health and sleep health within school and community settings [17,24]. Integrating digital use education into broader adolescent health promotion initiatives may help support balanced use patterns without framing smartphone engagement solely as a behavioral problem. These findings suggest the potential value of school-based digital hygiene programs and the integration of smartphone use monitoring into adolescent mental health screening frameworks.

This study has several strengths. It was based on a large, nationally representative sample of Korean adolescents, which enhances the generalizability of the findings within this population. The substantial sample size allowed stable estimation of associations across multiple sociodemographic and psychological factors. In addition, the inclusion of both conventional regression analyses and an exploratory modeling approach provided complementary perspectives on the observed associations, thereby strengthening the overall interpretation of the findings.

Several limitations should be acknowledged. First, the cross-sectional design precludes causal interpretation. The temporal direction of associations between smartphone use and psychological factors cannot be determined, and reciprocal relationships remain possible. Second, all variables were based on self-reported measures, which may be subject to recall bias or social desirability bias. Third, excessive smartphone use was operationalized using a time-based threshold (≥300 min per day). Although this threshold was informed by prior evidence suggesting potential deterioration in mental health outcomes beyond approximately five hours of daily screen exposure [28,29], it represents a pragmatic population-level indicator. It does not constitute a clinical diagnostic cutoff. Different thresholds could yield different prevalence estimates. Fourth, residual confounding by unmeasured factors cannot be excluded. Finally, the exploratory modeling analysis demonstrated only modest predictive discrimination, reflecting its supportive role rather than a primary predictive objective.

Although this study was conducted among Korean adolescents, the observed associations between developmental, socioeconomic, and psychological factors and excessive smartphone use are broadly consistent with findings reported in other adolescent populations. However, these patterns may vary depending on cultural norms, educational systems, and digital environments [5,17]. Therefore, caution is needed when generalizing the results, and further cross-cultural research is warranted.

## 5. Conclusions

Excessive smartphone use among Korean adolescents was associated with developmental, socioeconomic, psychological, and behavioral factors, including sex, school grade, perceived socioeconomic status, anxiety symptoms, sleep-related recovery, suicidal ideation, and vigorous physical activity. The complementary use of machine learning provided additional insights into the relative importance of factors associated with excessive smartphone use. The observed developmental and socioeconomic gradients suggest that patterns of excessive smartphone use vary across stages of adolescence and social contexts. From a pediatric public health perspective, these findings underscore the importance of developmentally informed and context-sensitive approaches to support balanced smartphone use and adolescent well-being. Given the use of nationally representative data, these findings may inform public health policies aimed at promoting balanced digital use among adolescents.

## Figures and Tables

**Table 1:** Sociodemographic, psychological, and behavioral characteristics of participants (N = 54,653).

Variable	Category	Value
Sex	Male	28,090 (51.4)
	Female	26,563 (48.6)
Grade	Grade 7	9887 (18.1)
	Grade 8	9725 (17.8)
	Grade 9	9475 (17.3)
	Grade 10	8972 (16.4)
	Grade 11	8719 (16.0)
	Grade 12	7875 (14.4)
Perceived SES	High	6330 (11.6)
	Upper-middle	16,815 (30.8)
	Middle	25,432 (46.5)
	Lower-middle	4980 (9.1)
	Low	1096 (2.0)
Perceived stress	Very high	6140 (11.2)
	High	16,902 (30.9)
	Moderate	22,963 (42.0)
	Low	7061 (12.9)
	Very low	1587 (2.9)
Suicidal ideation (yes)		6951 (12.7)
GAD-7 scores		11.5 ± 4.7
Fatigue recovery by sleep	Very sufficient	3763 (6.9)
	Sufficient	8565 (15.7)
	Neutral	16,574 (30.3)
	Insufficient	17,072 (31.2)
	Very insufficient	8679 (15.9)
Vigorous physical activity (days/week)		3.3 ± 2.2
Daily smartphone use (minutes)		304.2 ± 172.6

Note. Categorical variables are presented as *n* (%) and continuous variables as mean ± SD.

**Table 2:** Factors Associated with Excessive Smartphone Use Among Korean Adolescents (N = 54,653).

	B	S.E.	Wald	df	*p*-Value	Exp(B)	95% CI	
							Lower	Upper
Gender: female (reference: male)	0.365	0.019	356.745	1	<0.001	1.44	1.386	1.495
Grade 7 (reference)			631.537	5	<0.001			
Grade 8	0.374	0.031	145.678	1	<0.001	1.45	1.368	1.545
Grade 9	0.612	0.031	387.692	1	<0.001	1.84	1.735	1.960
Grade 10	0.470	0.032	212.465	1	<0.001	1.60	1.503	1.705
Grade 11	0.694	0.032	457.799	1	<0.001	2.00	1.878	2.133
Grade 12	0.662	0.033	391.724	1	<0.001	1.94	1.816	2.070
SES high (reference)			659.041	4	<0.001			
SES upper-middle	0.100	0.033	9.149	1	0.002	1.11	1.036	1.178
SES middle	0.418	0.031	177.126	1	<0.001	1.52	1.428	1.615
SES lower-middle	0.721	0.041	306.690	1	<0.001	2.06	1.897	2.229
SES low	1.067	0.074	209.521	1	<0.001	2.91	2.516	3.359
Perceived stress	−0.050	0.012	17.345	1	<0.001	0.95	0.929	0.974
GAD-7 total score	0.014	0.002	33.868	1	<0.001	1.01	1.009	1.019
Suicidal ideation (past 12 months)	0.180	0.030	36.121	1	<0.001	1.20	1.129	1.269
Perceived recovery from sleep	0.037	0.009	17.067	1	<0.001	1.04	1.020	1.056
Vigorous physical activity (days/week)	0.019	0.005	16.584	1	<0.001	1.02	1.010	1.029
Model fit: χ^2^ = 2478.95, df = 15, *p* < 0.001, explanatory power: Cox & Snell R^2^ = 0.046; Nagelkerke R^2^ = 0.062.								

CI = confidence interval, Exp(B) = adjusted Odds Ratio.

## Data Availability

The data analyzed in this study are publicly available from the Korea Youth Risk Behavior Web-based Survey (KYRBS), administered by the Korea Disease Control and Prevention Agency (KDCA). Data can be accessed upon registration at: https://www.kdca.go.kr/yhs/ (accessed on 11 November 2025).

## Supplementary Materials

The following supporting information can be downloaded at: https://www.mdpi.com/article/10.3390/children13040472/s1, Figure S1: SHAP summary plot; Figure S2: SHAP dependence plots.

## Abbreviations

The following abbreviations are used in this manuscript:

KYRBS	Korea Youth Risk Behavior Web-based Survey
KDCA	Korea Disease Control and Prevention Agency
LightGBM	Light Gradient Boosting Machine
SHAP	SHapley Additive exPlanations
AUC	Area Under the Curve
