# Supplementary material for: Psychosocial and Behavioral Factors Associated with Excessive Smartphone Use Among Korean Adolescents: A National Cross-Sectional Study
Source: Children (Basel). 2026 Mar 28;13(4):472. doi: 10.3390/children13040472 (PMC13114661; doi:10.3390/children13040472)
Supplement: Supplementary file 1 [file children-13-00472-s001.zip › children-4213206-Supplementary Materials.pdf]

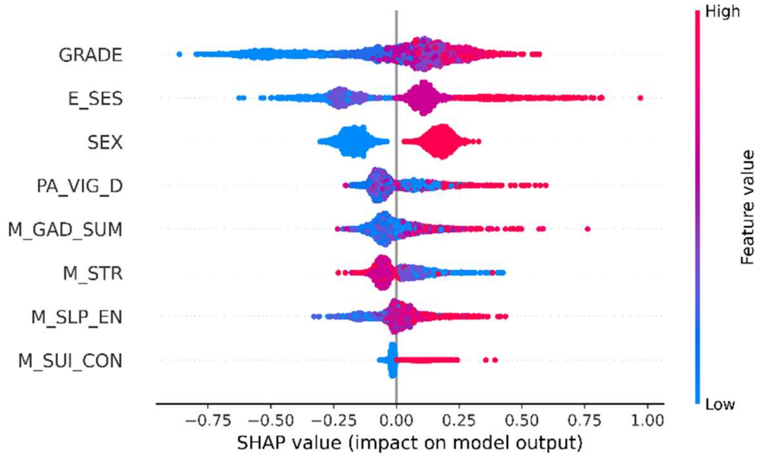

Supplementary Figure S1 SHAP summary plot showing the relative importance and direction of effects of predictors

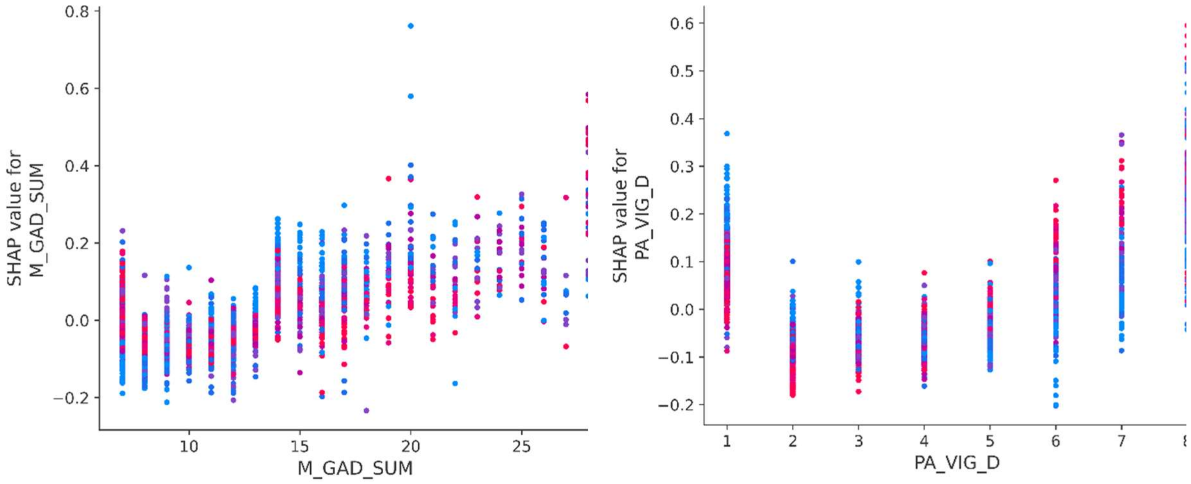

(**a**) (**b**)

Supplementary Figure S2 SHAP dependence plots for key behavioral and psychological predictors of smartphone overdependence.: (**a**) Generalized anxiety score (M_GAD_SUM) with grade as an interaction feature; (**b)** Days of vigorous physical activity (PA_VIG_D) with grade as an interaction feature.
